# Causes of mortality among new HIV-infected intravenous drug users in a clinical hospital in Bucharest

**DOI:** 10.1186/1471-2334-13-S1-O6

**Published:** 2013-12-16

**Authors:** Simona Erscoiu, Ionuț Popa, Denis Oncel, Olivia Burcoş, Maria Pătru, Emanoil Ceauşu

**Affiliations:** 1 Carol Davila University of Medicine and Pharmacy, Bucharest, Romania; 2 Clinical Hospital of Infectious and Tropical Diseases “Dr. Victor Babeş”, Bucharest, Romania

## Background

During the last 3 years, new routes of HIV infection have been reported in our country: addiction and homosexuality. Heroin use and the new ethnobotanic drugs (so called “legal drugs”) led to a dramatic increase in mortality in these intravenous drug users (IVDUs). A European multicentre study showed how IVDUs with recent HIV seroconversion are subject to an increased risk of mortality due to pneumonia, endocarditis, sepsis, meningitis, encephalitis, and decompensated liver cirrhosis. Before the availability of HAART, an increased rate of mortality due to heroin overdose and suicide was also recorded among HIV-infected IVDUs. Just the use of elevated heroin dosages associated to a suicidal behavior could be partially responsible for this phenomenon in patients with a newly diagnosed HIV disease and lacking social and psychological support, especially before the introduction of effective antiretroviral drug combinations (HAART).

The objective of our study was to evaluate the temporal trend of deaths in a cohort of IVDUs, newly infected with HIV and admitted to our clinic and to assess its relationship with HIV infection and AIDS, and availability of potent antiretroviral therapy.

## Methods

The investigation of the death condition of HIV infected IVDUs was conducted through a retrospective cohort study in the Clinical Hospital of Infection and Tropical Diseases “Dr. Victor Babeş”, Casa Andreea, from 01 January 2011 to 31 August 2013. A clinical and etiological analysis of different disorders was done. We excluded the patients missing to follow up and those who died at home.

## Results

Out of a total of 249 patients, 32 (13%) IVDUs died (mainly ethnobotanic addicts, 66%), 23 males and 9 females. The large majority of enrolled subjects were born in the Bucharest metropolitan area and surroundings. The mean age at death was 30 years old (limit 18-50). The median value of CD4 lymphocytes was 96/cmm. Most of the patients were unemployed and had no education (only primary school). The main death causes were as follows: AIDS (65.6%), ethnobotanic drug overdose (6.2%), sepsis with methicillin susceptible *Staphylococcus aureus* (37.5%), pulmonary or disseminated tuberculosis (28.1%), pneumonia (18.75%) and few cases of other diseases. Only one IVDU received antiretroviral treatment, without adherence.

## Conclusion

Stopping drug use, psychological counseling, early diagnosis of HIV status, counseling and social support, initiating tuberculosis and antiretroviral treatment, are some of the measures to be taken to decrease both the number of IVDUs and their death.

